# Emergent Revascularization After Transposition of an Unexpected Intraosseous Anomalous Subarcuate Loop During Vestibular Schwannoma Surgery: A Case Report

**DOI:** 10.1227/neuprac.0000000000000045

**Published:** 2023-06-12

**Authors:** Kiyohiko Sakata, Aya Hashimoto, Hidenobu Yoshitake, Sosho Kajiwara, Kimihiko Orito, Hideo Nakamura, Motohiro Morioka

**Affiliations:** * Department of Neurosurgery, Kurume University School of Medicine, Fukuoka, Japan;; ‡ Department of Pathology, Kurume University School of Medicine, Fukuoka, Japan

**Keywords:** Anomalous subarcuate loop, Anteroinferior cerebellar artery, Cerebellopontine angle, End-to-end anastomosis, Subarcuate fossa, Thrombectomy, Vestibular schwannoma surgery

## Abstract

**BACKGROUND AND IMPORTANCE:**

The presence of an anomalous anteroinferior cerebellar artery (AICA) embedded within the subarcuate fossa increases the difficulty of cerebellopontine angle (CPA) tumor surgery. Iatrogenic injury of posterior fossa arteries can result in serious morbidity.

**CLINICAL PRESENTATION:**

A 70-year-old man presented with right-sided hearing loss and facial dysesthesia. Magnetic resonance imaging showed a tumor with solid and cystic components and 35-mm maximum diameter in the right CPA. The AICA traveled just dorsal to the tumor and was well-developed because the ipsilateral vertebral artery and posteroinferior cerebellar artery (PICA) were aplastic. During surgery, we unexpectedly encountered an anomalous loop of the AICA-PICA which was embedded in the subarcuate fossa. This loop was mobilized using an ultrasonic bone curette to enable further tumor resection. However, it occluded immediately after mobilization and required open thrombectomy and end-to-end anastomosis. After revascularization, near-complete tumor resection was achieved without causing facial nerve dysfunction or brainstem/cerebellar infarction. Pathological examination of the resected anomalous loop showed abnormal focal hypertrophy of the adventitia and the presence of external elastic lamina.

**CONCLUSION:**

Mobilization of an anomalous AICA-PICA loop embedded within the subarcuate fossa during VS resection can result in arterial occlusion which requires thrombectomy and revascularization. Surgeons should be aware of this vascular anomaly and be prepared to deal with its ramifications.

**ABBREVIATIONS:**
H&E: hematoxylin & eosin
LA: labyrinthine artery
SAA: subarcuate artery
SL: subarcuate loop
VS: vestibular schwannoma.

Vestibular schwannoma (VS) is the most common tumor arising in the cerebellopontine angle (CPA). Although large VS represents a surgical challenge, maximal resection and facial nerve preservation remain the primary goals of surgery when surgical resection is selected.^1^

The anteroinferior cerebellar artery (AICA) gives rise to the subarcuate artery (SAA), which penetrates the dura along the posterosuperior wall of the internal acoustic meatus.^2,3^ Rarely, its origin along with a portion of the AICA can be embedded entirely within the dura or even within the petrous bone.^4-7^ The presence of such an anomalous AICA loop limits maneuverability during VS resection.^5-8^ Aplasia of the posteroinferior cerebellar artery (PICA) is another vascular anomaly occasionally encountered in which the AICA supplies most of the cerebellar hemisphere. The prevalence of this anomaly ranges between 20% and 24%.^9,10^

We present an instructive VS resection case in which an anomalous AICA-PICA loop was embedded in the subarcuate fossa and traversed just dorsal to the tumor. This unexpected anomalous loop required transposition to enable resection. However, after transposition, thrombosis occurred and urgent open thrombectomy was required.

## CLINICAL PRESENTATION

**FIGURE 1.**
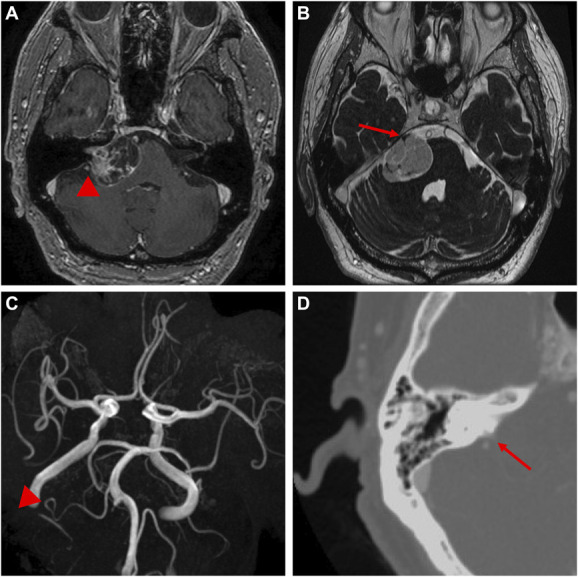
Preoperative magnetic resonance images. **A**, Gadolinium-enhanced T1-weighted imaging showed a right cerebellopontine angle tumor with enhancing solid and cystic components. A large vessel traverses the tumor dorsally (*arrowhead*). **B**, The trigeminal nerve was displaced anterosuperiorly on constructive interference in steady state imaging (*arrow*). **C**, Magnetic resonance angiography showed an aplastic right vertebral artery and PICA. A well-developed anteroinferior cerebellar artery PICA travels laterally and has an anomalous loop (*arrow*). **D**, Contrast-enhanced computed tomography showed a small dimple at the subarcuate fossa that contained a vessel (*arrowhead*). PICA, posteroinferior cerebellar artery.

A 70-year-old man presented with progressive right-sided hearing loss and facial dysesthesia. Hearing was nonserviceable on the right (Gardner-Robertson grade III). His medical history was unremarkable. There was no family history of neurofibromatosis type 2. Magnetic resonance (MR) images revealed a right CPA tumor with solid and cystic components compressing the brainstem and right trigeminal nerve. Maximum tumor diameter was 35 mm. The solid portion of the tumor exhibited contrast enhancement (Figure 1A and 1B). On MR angiography, the right vertebral artery and PICA were aplastic; the AICA was well-developed and traveled just dorsal to the tumor (Figure 1C). Contrast-enhanced computed tomography demonstrated a small dimple at the subarcuate fossa that contained a small vessel (Figure 1D). However, we did not recognize the implications of these findings before surgery. Surgical tumor resection through a lateral suboccipital retrosigmoid approach was planned (see Video).

**VIDEO.** Emergent revascularization after transposition of an unexpected intraosseous anomalous subarcuate loop during vestibular schwannoma surgery.

### Intraoperative Findings

**FIGURE 2.**
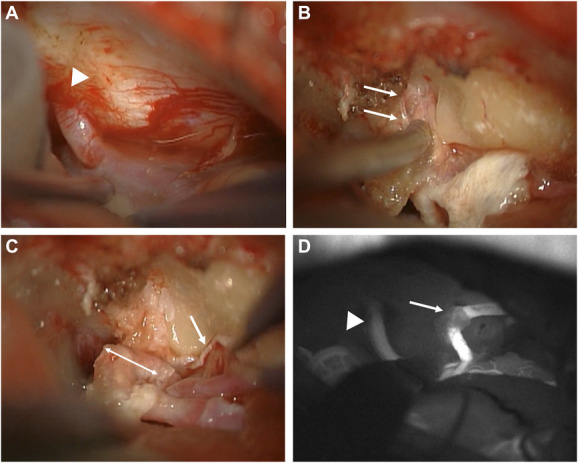
Intraoperative photography and indocyanine green video angiography. **A**, A common AICA-PICA trunk penetrated the dura of the subarcuate fossa (*arrowhead*). **B**, The tip of the anomalous subarcuate loop embedded in the subarcuate fossa gave rise to the subarcuate artery (*arrows*) inside the bone. **C**, The tip was embedded 5 mm deep within the bone (*double-headed arrow*). The anomalous loop was embedded at the posterosuperior wall of the internal acoustic meatus (*arrow*). **D**, Intraoperative indocyanine green video angiography showed arterial patency and smooth venous return to the superior petrosal vein (*arrowhead*) after end-to-end anastomosis of the AICA-PICA common trunk (*arrow*). AICA, anteroinferior cerebellar artery; PICA, posteroinferior cerebellar artery.

Surgery was performed with the patient in the park bench position under intraoperative facial nerve monitoring. A retroauricular lazy S incision was made, and a standard retrosigmoid craniotomy was performed. After exposing the CPA, 2 unanticipated anomalous arteries were discovered penetrating the dura in the subarcuate fossa (Figure 2A). Indocyanine green video angiography demonstrated that these arteries comprised a loop, namely the AICA-PICA common trunk. Although we reflected the dura of the subarcuate fossa to mobilize the loop, it ran deep into the petrous bone. Tumor extraction became difficult owing to its presence; therefore, the loop was transposed from the bone using an ultrasonic bone curette and dissector. During transposition, the SAA was visualized arising from the tip of the loop inside the bone (Figure 2B). The arterial loop embedded 5 mm deep within the bone was successfully transposed without injury (Figure 2C).

Unfortunately, micro-Doppler ultrasonography and indocyanine green video angiography after transposition showed occlusion of the arterial loop. We were unable to promote resumption of flow with arterial massage, topical application of papaverine hydrochloride, and aspiration; therefore, we performed open thrombectomy. A thrombus was removed from the distal stump of the artery after cutting the loop. Revascularization of the AICA-PICA common trunk was achieved with an end-to-end anastomosis (Figure 2D). Subsequently, tumor resection was completed without causing facial nerve dysfunction.

### Postoperative Course

**FIGURE 3.**
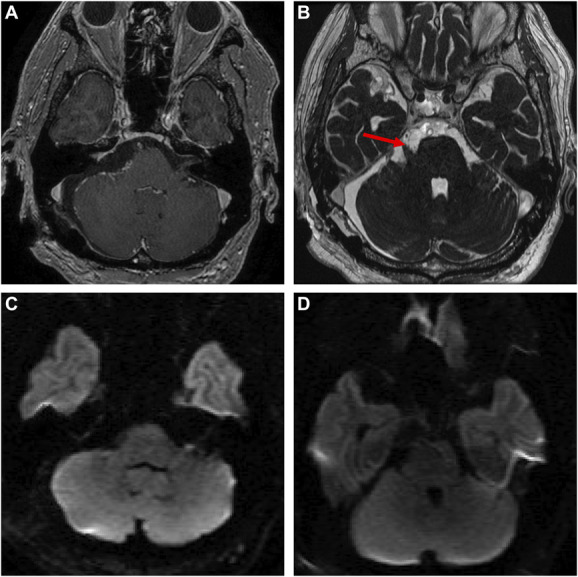
Postoperative magnetic resonance images. **A**, Gadolinium-enhanced T1-weighted imaging showed near-complete tumor resection and relief of brainstem compression. **B**, The trigeminal nerve was clearly visualized on constructive interference in steady state imaging (*arrow*). **C**, and **D**, Diffusion-weighted imaging showed no ischemic changes in the cerebellum or brainstem.

**FIGURE 4.**
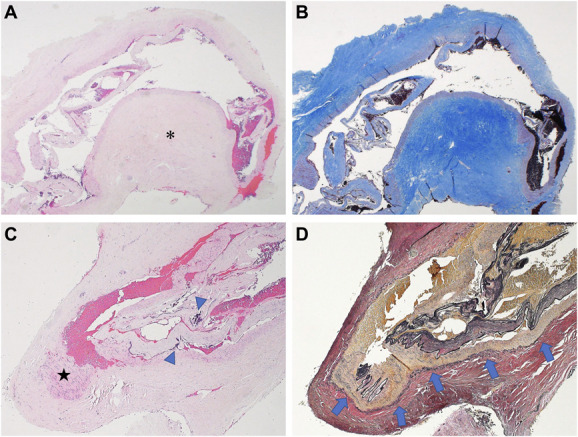
Pathological findings of the resected anomalous loop. **A**, Photomicrographs (H&E) showing focal hypertrophy of the adventitia (asterisk). **B**, This layer comprised numerous collagen fibers that were revealed by Masson trichrome staining. **C**, The vessel wall also showed fibroblast formation (star) and focal calcification (blue arrowheads) in the tunica media (H&E). **D**, Continuous elastic fibers were also seen between the tunica media and the tunica adventitia (blue arrows), which was revealed by Elastica van Gieson staining. These findings indicate that this anomalous loop comprised abnormal structures that differed from those in usual intracranial peripheral arteries. H&E, hematoxylin & eosin.

The patient's postoperative course was uneventful. Facial dysesthesia resolved fully, and facial nerve function remained normal (House and Brackmann grade I) after surgery. MR images showed near-complete tumor resection and no ischemic changes in the AICA-PICA territory (Figure 3). Pathological examination of the resected abnormal loop revealed focal fibrous hypertrophy of the adventitia and the presence of external elastic lamina (Figure 4). The patient is currently healthy without recurrence 4 years after surgery.

### Informed Consent

Informed consent was obtained from the patient. The patient consented to the publication of his image. Institutional ethics committee approval was obtained before submission.

## DISCUSSION

The SAA is an inconstant branch of the AICA or AICA-PICA which supplies the otic capsule of the semicircular canals, vestibule, facial nerve canal, and mastoid antrum.^11,12^ Although damage to the labyrinthine artery can result in hearing loss, SAA injury has no clear implications.^2,8,11^ In a cadaver study, SAA prevalence ranges from 35% to 85% and average length was 6.5 mm (range, 4-12).^2,11,13^ Therefore, the SAA can be safely coagulated and cut along the long stem between the AICA origin and subarcuate dura. However, the SAA is occasionally embedded within the subarcuate fossa to various degrees, sometimes even with the AICA or AICA-PICA itself. This anatomic variant has been called an “anomalous subarcuate loop (SL),”^4,7^ “AICA embedded in the subarcuate fossa,”^5,14^ “AICA penetrating the subarcuate fossa dura,”^6^ or “challenging AICA.”^8^

**FIGURE 5.**
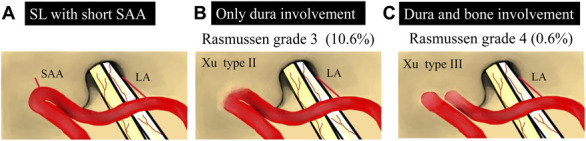
Illustrations of anomalous subarcuate loop. **A**, An anteroinferior cerebellar artery sometimes forms the convex curve directed toward the subarcuate fossa at the point where the subarcuate artery arose. **B**, The apex of the subarcuate loop can occasionally be adherent to the dura **C**, even be embedded in the bone. SAA, subarcuate artery; LA, labyrinthine artery.

In a MR imaging study of AICA-SAA variants, Rasmussen et al^15^ reported a 10.6% prevalence of “duralized” AICA with unidentifiable SAA or an SAA located within the petromastoid canal; the prevalence of intraosseous AICA with unidentifiable SAA or an SAA located within the petromastoid canal was 0.6% (Figure 5). The reported anomalous SL prevalence rates from surgical series ranges from 1.0% to 4.2%.^4,6-8^ In a series of 963 surgically treated VSs, Xu et al^7^ encountered 16 anomalous SLs. Among these, 7 were embedded in the bone. In the first 4, the tumor was removed without mobilization of the loop; in the last 3, the tumor was removed after releasing the anomalous SL. Although preoperative diagnosis of an anomalous SL remains difficult, the “deep subarcuate fossa” sign, which is a dimple of the petrous bone exceeding 2 mm in depth, may be a radiological feature that indicates an anomalous SL embedded in the bone.^7^

Several authors have reported techniques for transposing an anomalous SL during CPA surgery.^4-8,14,16-18^ However, Tanriover and Rhoton argued that transposition places the AICA at a risk of bleeding and/or occlusion from thrombosis or spasm.^5^ Furthermore, they noted that unexpected AICA bleeding may occur when a transpetrosal approach is used owing to the limited or lack of proximal arterial control. In our patient, arterial thrombosis occurred despite the absence of direct vascular injury during transposition. We argue that even careful maneuvers during transposition of such abnormal vessels carry a risk of inducing thrombus formation.^19,20^ Although brainstem and cerebellar ischemia associated with perforating vessels after VS surgery are often asymptomatic,^21^ we chose open thrombectomy and end-to-end anastomosis for revascularization because serious cerebellar infarction with a wide territory was likely after occlusion of the AICA-PICA common trunk.^22,23^ Endovascular thrombectomy was another consideration^24,25^; however, this therapy may be unsuitable for this distal loop. Fellow surgeons should be aware of the anomalous SL when performing VS resection and be prepared to deal with its ramifications.

### Limitations

Fortunately, we successfully addressed this dangerous situation; however, this patient would have been exposed to a critical risk of developing neurological deficits if the revascularization procedures were unsuccessful. A courageous decision to leave the tumor and pursue radiosurgery should also be allowed.^26-28^

## CONCLUSION

Mobilization of an anomalous AICA-PICA loop embedded within the subarcuate fossa during VS resection can result in arterial occlusion which requires thrombectomy and end-to-end anastomosis. Surgeons should be aware of this vascular anomaly and be prepared to deal with its ramifications.

## COMMENTS

Preparation is a key aspect of surgery: considering the technical and anatomical aspects as well as complication responses preoperatively will make for a safer and more efficient surgery. In this report, the authors present a case of intraoperative microvascular occlusion of the AICA during resection of a vestibular schwannoma. They discuss their response to this occlusion by opening the vessel, performing open thrombectomy, and end-to-end anastomosis. Revascularization in this case was necessary as it was a common origin AICA-PICA branch, as well as the origin of the subarcuate artery.

This case underscores the importance of high attention to detail, specifically in identifying the issue after transposition of the vessel loop overlying the tumor, with the use of Doppler and fluorescence. Had this not been identified at that time, the downstream consequences postoperatively may have been grave. This case elegantly shows the role of preoperative planning and in-depth analysis of the imaging, planning for complications, and intraoperative attention to the entire field, not just focus on the end goal (ie, tumor resection).

**Kurt Yaeger**

*Houston, Texas, USA*
